# Red Breast Syndrome and Early Postoperative Complications in Immediate Prepectoral Implant-Based Reconstruction Using Braxon® Acellular Dermal Matrix: A Retrospective Cohort Study

**DOI:** 10.7759/cureus.104536

**Published:** 2026-03-02

**Authors:** Rami Oweis, Shadan M Jan, Dominic Luttrell, Bertha In 't Hout, Lisa Caldon, Tahir Masudi

**Affiliations:** 1 Breast Surgery, The Rotherham NHS Foundation Trust, Rotherham, GBR; 2 Internal Medicine, Imperial College Healthcare, London, GBR; 3 Medicine and Surgery, The Rotherham NHS Foundation Trust, Rotherham, GBR; 4 General Surgery, Bradford University Hospital, Bradford, GBR

**Keywords:** adm, breast cancer, breast reconstruction, mastectomy, red breast syndrome

## Abstract

Background: Red breast syndrome (RBS) is a self-limited, non-infectious erythema historically linked to acellular dermal matrices (ADMs) in breast reconstruction. Recent reports suggest a declining incidence, potentially related to ADM advancements. This study evaluates the contemporary incidence of RBS and postoperative complications in patients undergoing immediate prepectoral reconstruction with a specific porcine ADM (Braxon®, Decomed s.r.l., Venice, Italy).

Methods: This is a retrospective cohort study of all consecutive patients who underwent immediate prepectoral breast reconstruction with ADM (Braxon®) at our institution. Patients were identified from a prospectively maintained database. The study included all procedures performed between August 1, 2020, and October 31, 2024 (the data cutoff date for analysis). Data on demographics, surgical details, and postoperative outcomes were collected. Complications were classified as minor (managed conservatively) or major (requiring reoperation). RBS was defined by strict clinical criteria excluding infection. Univariate analysis assessed risk factors for complications.

Results: No cases of RBS were observed (0/95, 0%). Early minor complications included seroma (7/95, 7.3%), skin necrosis (4/95, 4.2%), and infection (1/95, 1%). Major complications requiring reoperation occurred in 15.8% (15/95) of cases, with an overall implant loss rate of 13.7% (13/95). Preoperative radiotherapy significantly increased early complications (RR = 3.2, p = 0.013) and implant loss risk six-fold (RR = 6.17, p = 0.007). Postoperative radiotherapy was associated with higher capsular contracture rates (RR = 3.73, p = 0.008). Smoking, necrosis, infection, and dehiscence were significant predictors of implant loss (p < 0.05).

Conclusion: Notably, no cases of RBS were identified in this cohort, supporting the safety of Braxon® ADM in prepectoral reconstruction and contributing to evidence of its declining incidence. This absence may be attributed to specific ADM properties, surgical technique, and patient selection. However, radiotherapy and smoking remain critical, modifiable risk factors for implant-related complications.

## Introduction

Red breast syndrome (RBS) is a postoperative phenomenon characterized by blanching erythema of the reconstructed breast in the absence of infection [1]. It was first described in 2009 by Nahabedian in patients undergoing submuscular breast reconstruction with acellular dermal matrix (ADM) [2]. RBS usually develops days to weeks following surgery and resolves spontaneously within weeks to months without treatment, therefore being self-limited [1,3].

Although the clinical presentation can mimic cellulitis and often prompts empiric antibiotic therapy [3,4], the condition is not associated with significant pain, increased skin temperature, or palpable induration [3,4]. Additionally, laboratory indicators of infection, such as elevated white blood cell count, C-reactive protein, and erythrocyte sedimentation rate, are typically within normal limits [3]. The etiology of RBS remains unclear [3,4], is likely multifactorial [3], and currently, there is no established protocol for treatment [3].

Multiple hypotheses have been proposed to explain the pathophysiology of RBS, including dependent erythema due to disrupted lymphatic flow [3], immune or allergic mechanisms [3], prior radiotherapy [3] or chemotherapy [3], and cutaneous hyperemia from vascular ingrowth into the ADM [1]. The most widely believed hypothesis implicates an association with the use of ADMs [3,5-8], as erythema often localizes to areas where the ADM has been placed [2], suggesting an inflammatory response to the matrix material [3,5,9,10]. Reports also exist of similar erythema with synthetic meshes [11-13].

Historically, RBS has been reported with various ADM types [3]. However, recent meta-analyses indicate a potential decline in its incidence, with a weighted mean of 3.22% reported for studies published between 2009 and 2023 [4], compared to 6.4% in a 2018 analysis [4]. While suggestive, this comparison is limited by the overlapping study periods and does not necessarily represent a true chronological trend. This decline may reflect improvements in ADM processing (such as Braxon®, Decomed s.r.l., Venice, Italy), surgical techniques (such as the shift to the prepectoral plane), or refined patient selection.

This study aimed to evaluate the contemporary incidence of RBS and other postoperative complications in a consecutive cohort undergoing immediate prepectoral breast reconstruction with a specific non-crosslinked porcine ADM (Braxon®) [14-17].

## Materials and methods

We conducted a retrospective cohort study of all consecutive patients who underwent immediate prepectoral breast reconstruction with ADM (Braxon®) at our institution. Patients were identified from a prospectively maintained database. The study included all procedures performed between August 1, 2020, and October 31, 2024 (the data cutoff date for analysis). The work has been reported in line with the STROCSS (Strengthening the Reporting of Cohort, Cross-Sectional, and Case-Control Studies in Surgery) criteria [18]. Inclusion criteria consisted of patients undergoing immediate, prepectoral implant-based breast reconstruction using Braxon® ADM. Exclusion criteria included delayed reconstructions, revisions, autologous reconstructions, or procedures using other ADMs/synthetic meshes. Patients with less than three months of follow-up were excluded from complication analysis. At our institution, inclusion criteria did not exclude patients based on smoking status; consequently, both smokers and non-smokers were enrolled in the cohort. The absence of significant comorbidities that would severely compromise wound healing (e.g., uncontrolled diabetes) was also excluded.

Subcutaneous mastectomies were performed by specialist breast oncoplastic surgeons, preserving well-vascularized mastectomy flaps. Immediate prepectoral reconstruction was performed using Braxon® ADM, a 0.6 mm-thick, non-crosslinked collagen matrix derived from porcine dermis. Braxon® Fast is its pre-shaped, three-dimensional counterpart with identical biological properties. The technique followed previously published protocols [14-16]. Briefly, the ADM was hydrated in saline and sutured around the implant with 2-0 polydioxanone (PDS) to create a complete biologic envelope. The ADM-implant construct was then placed in the prepectoral pocket and secured to the pectoralis fascia and subcutaneous tissue. All patients received intravenous amoxicillin/clavulanate at induction and five days of oral amoxicillin/clavulanate postoperatively.

RBS was defined a priori as the presence of a well-demarcated, blanchable erythema overlying the ADM, occurring in the postoperative period, in the absence of criteria for infection. Diagnostic criteria for exclusion of infection included the following: no systemic signs (fever >38°C), no purulent discharge, normal or minimally elevated inflammatory markers (white blood cell count <11 x10³/µL; C-reactive protein <10 mg/L), and negative culture from any aspirated fluid from the reconstructed cavity. Cases of erythema were evaluated clinically at each follow-up, and any suspicion of infection prompted appropriate investigation.

Demographic, surgical, and postoperative data were collected. Early complications were defined as those occurring within three months of surgery. Complications were classified as minor (managed conservatively in an outpatient setting, e.g., seroma aspirated in clinic and oral antibiotics for minor cellulitis) or major (required a return to the operating theatre, e.g., surgical debridement, implant washout, or explantation). Long-term follow-up assessed capsular contracture graded by the Baker classification.

Continuous variables are presented as mean ± standard deviation or median (range). Categorical variables are presented as frequencies and percentages. Univariate analysis was performed to identify associations between risk factors and complications. Relative risk (RR) with 95% confidence intervals was calculated using the χ² test or Fisher's exact test, as appropriate. A p-value of <0.05 was considered statistically significant. Analyses were performed using SPSS Statistics version 28 (IBM Corp., Armonk, NY).

## Results

Between August 2020 and October 2024, 107 patients underwent breast reconstruction. Of these, 78 patients (95 breasts) met the inclusion criteria. Demographic and surgical characteristics are detailed in Table 1. The mean age was 52.9 years (range = 32-76 years), and the mean BMI was 28.5 ± 5.6 kg/m². Most patients were non-smokers (87/95, 91%). Comorbidities included vascular conditions (17/95, 17.9%), hypertension (20/95, 21.1%), and diabetes (5/95, 5.3%). Surgery was unilateral in 58 patients (77.3%) and bilateral in 17 (22.7%). The primary indication was oncological for 76 breasts (80%), including ductal carcinoma in situ (DCIS, n = 30), invasive carcinoma (n = 41), and lobular carcinoma in situ (LCIS, n = 5). Nipple-sparing mastectomy was performed in 56 breasts (58.9%). Neoadjuvant and adjuvant chemotherapy were administered in 29.5% (28/95) and 39.7% (37/93) of cases, respectively. Radiotherapy was delivered preoperatively in four breasts (4.2%) and postoperatively in 25 breasts (26.3%). The mean implant volume was 423 cc. The mean follow-up was 20.1 months (range = 3-48 months).

**Table 1:** Demographic data and surgical details. SD: standard deviation; BMI: body mass index (measured in kg/m²); BRCA1/2mut: carriers of a BRCA1 or BRCA2 gene mutation; DCIS: ductal carcinoma in situ; LCIS: lobular carcinoma in situ; IDC: invasive ductal carcinoma; ILC: invasive lobular carcinoma; COPD: chronic obstructive pulmonary disease; gr: grams; cc: cubic centimeters.

Demographics	Number
Patients​	78
Breasts​	95
Follow-up (months) (Mean ± SD)​	20.14 ± 12.1 (3-48)
Age​ (years) (Mean ± SD)​	52.9 (32-76)
BMI (kg/m^2^) (Mean ± SD)​	28.55 ± 5.6
Hospital stay​ (days) (Mean ± SD)​	1.1 ± 0.6
Smoking status (per patient)	
Non-smokers	71 (91%)
Current smokers	5 (6.4%)
Former smokers	2 (2.6%)
Comorbidities (per patient)	
No	39 (50%)
Yes	39 (50%)
Diabetes	3 (3.8%)
Vascular diseases	14 (17.9%)
Other comorbidities (hypertension, COPD, dyslipidemia)	36 (46.2%)
BRCA1/2^mut^ carriers	17 (17.9%)
Surgery (per breast)	
Therapeutic	71 (74.7%)
Risk-reducing	24 (25.3%)
Unilateral	61 (78.2%)
Bilateral	17 (21.8%)
Type of tumor (per breast)	
DCIS	12 (12.6%)
LCIS	0
IDC	14 (14.7%)
ILC	9 (9.5%)
Mixed	35 (36.8%)
Other	6 (6.3%)
Mastectomy (per breast)	
Nipple-sparing	56 (58.9%)
Skin-sparing	38 (40%)
Skin-reducing	1 (1.1%)
Incisions (per breast)	
Batwing	4 (4.2%)
Hockey stick	39 (41%)
Wise pattern	39 (41%)
Inframammary fold	7 (7.3%)
Inframammary fold-lateral	1 (1%)
Lateral	1 (1%)
Lollipop	2 (2.1%)
Vertical	2 (2.1%)
Therapies	
Chemotherapy (per patient)	
Neoadjuvant	23 (29.5%)
Adjuvant	31 (39.7%)
Radiotherapy (per breast)	
Pre-operative	4 (4.2%)
Post-operative	25 (26.3%)
Other details (per breast)	
Breast weight (grams) (Mean ± SD)​	610.4 gr ± 369.7 gr
Implant volume (cc) (Mean ± SD)​	423.4 cc ± 114.4 cc
Axillary lymphadenectomy	15 (23,4%)
Previous breast surgery	26 (33.3%)

No cases of RBS were observed (0/95 breasts, 0%). Complication rates are summarized in Table 2, with early minor complications occurring in 14.7% (14/95) of breasts, most commonly seroma (7/95, 7.3%). Major complications requiring reoperation occurred in 15 breasts (15.8%), resulting in an overall implant loss rate of 13.7% (13/95). During long-term follow-up, capsular contracture was observed in 14 breasts (14.7%): one (1%) developed Baker grade III, and 13 (13.7%) developed Baker grade II. The single Baker III case occurred in a patient who had received postmastectomy radiotherapy.

**Table 2:** Complications.

Early complications	
Seroma	7 (7.3%)
Infected seroma	2 (2.1%)
Skin necrosis	4 (4.2%)
Red breast syndrome	0
Dehiscence	1 (1%)
Infection	4 (4.2%)
Skin necrosis	5 (5.2%)
Wound dehiscence	2 (2.1%)
Hematoma	1 (1%)
Other	2 (2.1%)
Late complications	
Capsular contracture	
Baker IV	0
Baker III	1 (1%)
Baker II	13 (13.7%)
Baker I	81 (85.3%)
Implant loss	13 (13.7%)

Univariate analysis of risk factors is shown in Table 3. Analysis of therapy-related risk factors for early postoperative complications identified a significantly increased risk in patients who underwent preoperative radiotherapy (RR = 3.2, 95% CI = 1.23-8.32; χ² = 6.10, p = 0.013). Capsular contracture (Baker grades II and III) was significantly more likely in patients who received postoperative radiotherapy (RR = 3.73, 95% CI = 1.33-10.43; χ² = 7.06, p = 0.008). Regarding implant loss, statistically significant associations were found with smoking status (RR = 4.87, 95% CI = 1.18-20.11; Fisher's exact p = 0.027), preoperative radiotherapy (RR = 6.17, 95% CI = 1.52-25.01; Fisher's exact p = 0.007), the development of necrosis (RR = 5.97, 95% CI = 1.90-18.76; χ² = 11.56, p < 0.001), infection (RR = 6.59, 95% CI = 2.02-21.52; χ² = 10.48, p = 0.001), and wound dehiscence (RR = 5.4, 95% CI = 1.30-22.38; Fisher's exact p = 0.015).

**Table 3:** Univariate analysis associating risk factors to early complications, capsular contracture, and implant loss. RR: relative risk; CI: confidence interval; CT: chemotherapy; RT: radiotherapy; *: p-value < 0.05. All values with a statistically significant p-value (p < 0.05) are highlighted in bold.

Outcome & predictors	RR	95% CI	p-value
Early complications			
Neo-adjuvant CT	1.32	0.58 - 3.00	0.5
Adjuvant CT	0.94	0.40 - 2.20	0.97
Pre-op RT	3.25	1.28 - 8.24	0.013*
Post-op RT	1.10	0.45 - 2.70	0.96
Capsular contracture (Baker II and III)			
Neo-adjuvant CT	1.49	0.46 - 4.80	0.51
Adjuvant CT	1.55	0.59 - 4.05	0.37
Pre-op RT	0	-	1
Post-op RT	3.73	1.41 - 9.87	0.008*
Implant loss			
Smoke	4.87	1.20 - 19.70	0.027*
Previous surgery	1.42	0.19 - 10.44	0.73
Neoadjuvant CT	1.19	0.15 - 9.32	0.97
Adjuvant CT	0.38	0.07 - 2.12	0.27
Pre-op RT	6.17	1.64 - 23.18	0.007*
Post-op RT	0.51	0.06 - 4.15	0.53
Necrosis	5.97	2.45 - 14.56	0.00009*
Seroma	1.26	0.20 - 7.79	0.9
Infection	6.59	2.11 - 20.58	0.001*
Dehiscence	5.4	1.38 - 21.08	0.015*

## Discussion

This study reports a notable finding: a complete absence of RBS in a contemporary series of 95 immediate prepectoral breast reconstructions using Braxon® ADM. This 0% incidence contrasts with the historical weighted mean of 3.22-6.4% reported in the literature [4,6] and aligns with a recent systematic review noting a declining trend in RBS reports [6]. Our complication profile otherwise aligns with known risks, particularly the profound impact of radiotherapy [1,2], affirming the generalizability of our cohort for complication assessment.

The absence of RBS is a pivotal observation. Several factors may contribute to this result. First, ADM-specific properties may be crucial. Braxon® is a thin (0.6 mm), non-crosslinked porcine dermal matrix. Its minimal thickness and lack of chemical cross-linking may facilitate rapid host cell infiltration and revascularization, potentially reducing the prolonged inflammatory phase hypothesized to cause RBS [1,3,5]. This is supported by comparisons showing variable complication profiles between different ADMs. Second, the prepectoral surgical plane may play a role. By avoiding submuscular dissection, this technique potentially preserves the lymphatic drainage of the mastectomy flap more effectively than the dual-plane technique, mitigating the "dependent erythema" related to lymphatic disruption [8,9]. Third, refined patient selection, requiring good-quality mastectomy flaps and abstention from smoking, likely created an optimal host environment for ADM integration, factors known to influence infection and inflammatory risks [8]. Finally, the application of strict diagnostic criteria for RBS, rigorously excluding low-grade infections or other causes of erythema like eosinophilic dermatoses through clinical and biochemical assessment, may prevent over-diagnosis and align with modern, precise definitions of the syndrome.

Our overall major complication (15.8%, 15/95) and implant loss (13.7%, 13/95) rates are slightly higher than some historical cohorts. This is readily explained by our cohort's significant proportion of patients receiving radiotherapy (30.5% combined pre- and postoperative), a known potent risk factor [1,2]. Our data powerfully quantify this risk: preoperative radiotherapy increased early complications three-fold and implant loss six-fold, while postoperative radiotherapy nearly quadrupled the risk of capsular contracture. These findings identify radiotherapy as the predominant risk factor in implant-based reconstruction; however, its administration is frequently mandated by oncologic considerations, limiting its modifiability. Other identified risk factors for implant loss, including active smoking, skin necrosis, infection, and dehiscence, are well-established in the literature [12,15,17] and highlight the paramount importance of viable wound closure.

Despite a high rate of postoperative radiotherapy (26.3%, 25/95), the incidence of severe (Baker III) capsular contracture was low at 1% (1/95). This is encouraging and suggests that the ADM's role as a regenerative, bioactive interface may exert a protective effect against the pro-fibrotic stimulus of radiation, even if milder (Baker II) contracture remains common.

This study has several limitations inherent to its retrospective, single-center design. While the cohort is substantial for an evaluation of a specific ADM, the sample size may limit the power to detect very rare events or subtle risk factor associations. Furthermore, the mean follow-up of 20.1 months, while adequate for assessing early and mid-term complications like RBS and implant loss, is relatively short for the definitive evaluation of long-term outcomes such as capsular contracture and implant durability. Future prospective, multi-center studies with longer follow-up are needed to validate these findings.

## Conclusions

In this contemporary cohort, the complete absence of RBS (0/95, 0%) suggests that modern, thin, non-crosslinked porcine ADMs (Braxon®) combined with a prepectoral approach may be associated with a reduced risk of this historically troubling complication, although this observation is limited by the retrospective design and sample size. This finding supports the safety profile of this specific ADM in immediate prepectoral reconstruction. The study reaffirms that radiotherapy remains the most significant predictor of adverse outcomes, drastically increasing the risks of early complications, implant loss, and capsular contracture. These results contribute to the evolving understanding of ADM-related complications and underscore the importance of meticulous patient selection and technique optimization.
